# Characterizing patients with rare mucormycosis infections using real-world data

**DOI:** 10.1186/s12879-022-07115-w

**Published:** 2022-02-14

**Authors:** Yayue Zhang, Anita H. Sung, Emily Rubinstein, Michael Benigno, Richard Chambers, Nataly Patino, Jalal A. Aram

**Affiliations:** 1 Hematology and Oncology Department, Dongzhimen Hospital, Beijing University of Chinese Medicine, Hai Yun Cang on the 5th Zip, Dongcheng District, Beijing, China; 2 Pfizer Inc, Hospital BU, 23 East 42nd Street, New York, NY 10017 USA

**Keywords:** Invasive mucormycosis, Zygomycosis, Electronic health records, Hematologic malignancies, Epidemiology, Real-world data

## Abstract

**Background:**

Invasive mucormycosis (IM) is a rare and often life-threatening fungal infection, for which clinical and epidemiological understanding is lacking. Electronic health record (EHR) data can be utilized to elucidate large populations of patients with IM to address this unmet need. This study aimed to descriptively assess data on patients with IM using the Optum® EHR dataset.

**Methods:**

US patient data from the Optum® deidentified EHR dataset (2007–2019) were analyzed to identify patients with IM. Patients with hematologic malignancies (HM), at high risk of IM, were selected and sorted by IM diagnosis (ICD9 117.7; ICD10 B46). Demographics, comorbidities/other diagnoses, and treatments were analyzed in patients with IM.

**Results:**

In total, 1133 patients with HM and IM were identified. Most were between 40 and 64 years of age, Caucasian, and from the Midwest. Essential primary hypertension (50.31%) was the most common comorbidity. Of the 1133 patients, only 33.72% were prescribed an antifungal treatment. The most common antifungal treatments were fluconazole (24.27%) and posaconazole (16.33%), which may have been prophylactic, and any AmB (15.62%).

**Conclusions:**

A large population of patients with IM were identified, highlighting the potential of analyzing EHR data to investigate epidemiology, diagnosis, and the treatment of apparently rare diseases.

## Background

Invasive mucormycosis (IM) is a rare and life-threatening invasive fungal infection caused by fungal species of the order Mucorales [1–3]. Mortality of patients with IM has been observed to range from 20–78%, although the small number of patients in these studies limits the reliability of these results [4, 5]. The term ‘zygomycosis’ is often used synonymously with mucormycosis, but also includes infections with species of the order Entomophthorales [3]. IM diagnoses are categorized, according to the European Organization for Research and Treatment of Cancer/Mycoses Study Group, as ‘proven’, ‘probable’, or ‘possible’ [6]. ‘Proven’ IM is diagnosed with certainty by the established presence of fungi; ‘probable’ IM is suggested by the presence of host factor criteria, clinical features, and mycological evidence; ‘possible’ IM is suggested by the presence of only host factor criteria [7]. Diagnostic techniques include radiological procedures (for example, chest and cranial CT scans and cranial MRIs), microbiological procedures (including quantitative PCR, high-resolution melting, and multiplex targeting) and histological procedures (including biopsies, direct microscopy using fluorescent brightener, and histopathology with stains such as periodic acid–Schiff and Grocott-Gomori methenamine silver stain) [8, 9].

Diagnosis of IM through these procedures is difficult, because fungal elements may not be detected in biopsies due to unviable fungal elements in homogenized tissues and, if present, may be fragmented in patient samples [8]. Additionally, clinical, radiographic, and cytological procedures have limited sensitivity, and the time taken to produce results can delay diagnosis [10]. As a result, diagnosis by autopsy or in the 24 h before death has been reported in around 10–46% of cases when studied [8, 11]. Diagnosis at a late stage of disease progression can delay treatment, leading to an increased risk of treatment failure and increased mortality [8, 12]. Although molecular diagnostic procedures are available, radiographic and biopsy sampling diagnoses are still often used [10].

The majority of patients with IM are immunocompromised, and previous studies have shown that 44–62% of patients have hematologic malignancies (HMs) [11, 13]. Previous epidemiological studies in patients with IM identified acute myeloid leukemia (48–51% of patients) and acute lymphoblastic leukemia (22–27% of patients) as the most commonly observed malignancies [8, 11]. Treatment recommendations for IM include liposomal amphotericin B (AmB) or, in the case of pre-existing renal compromise, intravenous isavuconazonium sulfate or posaconazole [9], as well as prophylactic treatment with azoles in neutropenic patients and surgical resection in combination with the last known effective treatment in immunosuppressed patients with a prior IM diagnosis [14]. Surgery in combination with antifungal treatment, most commonly 5–10 mg/kg/day liposomal AmB, is strongly recommended as a first-line therapy in adult patients with IM [9, 14].

Due to the rarity of IM, clinical, epidemiological, and outcomes data are lacking and are largely limited to case studies [1, 2, 15]. Large administrative datasets, for example claims databases and electronic health records (EHRs), are increasingly being used to facilitate analyses across many therapy areas [16]. Analysis of large datasets can be applied to many aspects of healthcare research, including epidemiology, pharmacovigilance, clinical trial recruitment, health economics and outcomes research, drug discovery, and diagnostics [16–18].

One example of a large dataset is the Optum® deidentified EHR dataset (2007–2019), which contains data from approximately 97 million patients from the USA. These data provide the opportunity to assess the population size and characteristics of patients with IM in a real-world setting, within the general population. This study aimed to descriptively assess data on patients with IM using the Optum® EHR dataset. It is hoped that characterizing a large pool of patient data will facilitate comparisons between real-world data and existing case studies, which will improve the diagnosis and treatment of this rare and life-threatening infection in the coming years.

## Methods

### Study data

This retrospective, observational study was conducted using patient data from the Optum® deidentified EHR dataset (2007–2019). The Optum® data acquisition model aggregates deidentified EHR data from providers across the continuum of care.

Optum®’s longitudinal EHR repository is derived from dozens of healthcare provider organizations in the United States that include more than 700 hospitals and 7000 clinics, and treat more than 103 million patients receiving care. The data are certified as deidentified by an independent statistical expert following Health Insurance Portability and Accountability Act statistical deidentification rules, and managed according to Optum® customer data use agreements [19, 20]. Clinical, claims, and other medical administrative data are obtained from both inpatient and ambulatory EHRs, practice management systems, and numerous other internal systems. Information is processed, normalized, and standardized across the continuum of care from both acute inpatient stays and outpatient visits. Optum® data elements include: demographics, medications prescribed and administered, immunizations, allergies, laboratory results (including microbiology), vital signs and other observable measurements, clinical and inpatient stay administrative data, and coded diagnoses and procedures.

### Patient selection

**Fig. 1:**
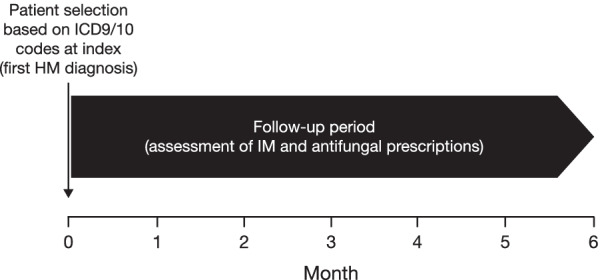
Study design. *HM* hematologic malignancies, *IM* invasive mucormycosis

Patients (male or female of any age) with any diagnosis of HM, who would be in the high IM risk category, were included in the analyses to reduce the possibility of false IM coding. International classification of diseases, ICD9 (pre-October 2015) and ICD10 (from October 2015), codes were then used to group patients into the following sub-evaluation cohorts: acute myeloid leukemia (AML), other lymphomas, other leukemias and other HM, as well as patients with neutropenia and stem cell transplants. ICD codes (ICD9 117.7; ICD10 B46.x) were then used to identify patients with IM from the HM cohort. Note that the method of diagnosis could not be retrospectively confirmed from deidentified patient data. The index date was the diagnosis of HM, and the observation period was at least 6 months of follow-up, including the assessment of IM (Fig. 1).

Among the overall HM cohort, patients with invasive aspergillosis (IA) infection (ICD9 117.3, 117.9, 484.6, 518.6; ICD10 B44.0, B44.1, B44.7) or coinfection with IM and IA within 360 days post-index were also explored. Treatment outcomes and mortality rates were not available in the dataset.

### Statistical analyses

Descriptive statistics were used to analyze demographics, comorbidities, and other diagnoses (including mean Charlson Comorbidity Index [CCI] score [21, 22]) and treatments in the patient sample. No statistical hypotheses were tested.

## Results

### Patient demographics and clinical characteristics

Approximately 100 million patients were included in the Optum® EHR dataset at the time of the analysis. Of these, 962,428 patients had an HM, and 1133 (0.001% of the overall patient population, 0.12% of HM) had an IM diagnosis (Table 1).

**Table 1:** Cohort selection steps from the Optum® EHR dataset

Group selection steps	Patients	% of total patients	% of patients in step 2 (1)
1. All the patients enrolled during Jan 2007–Jun 2019 in Optum®	101,340,454	100.00	–
2. (1) and with HM and 6 months follow-up	962,428	0.95	100.00
3. (2) and with IA between index and enrollment end date	10,638	0.01	1.11
4. (2) and with IM between index and enrollment end date	1133	0.00	0.12
5. (2) and coinfected with IA and IM within 360 days post-index	161	0.00	0.02

(1) Refers to the overall total number of 101,340,454 patients

(2) Refers to the 962,428 patients with HM and 6 months follow-up

*EHR* electronic health record, *HM* hematologic malignancy, *IA* invasive aspergillosis, *IM* invasive mucormycosis

In the 1133 patients with HM and IM, diagnosis of IM occurred after the diagnosis of HM in 95.94% of patients. HM and IM were diagnosed on the same day in the remaining 4.06% of patients. Of the 962,428 patients with HM, a total of 10,638 (1.11%) were diagnosed with IA. There were 161 patients in the HM cohort coinfected with IA and IM (14.21% of all patients with HM and IM).

Among the patients with HM and IM, the ICD9/ICD10 HM diagnoses were neutropenia (332 patients, 29.30%), leukemia (208 patients, 18.36%), lymphoma (165 patients, 14.56%), AML (145 patients, 12.80%), stem cell transplant (106 patients, 9.36%), and other HM (102 patients, 9.00%). Among patients with HM and IM with index events during the years 2007 through 2019, about one-fifth of IM cases identified occurred during each of the years 2015, 2016, and 2017, whereas fewer cases were identified in earlier years.

In the overall HM cohort, most patients were 40–64 years of age (374,517 patients, 38.91%) or > 65 years of age (355,453 patients, 36.93%), and the majority were Caucasian (757,088 patients, 78.66%) and from the Midwest region (505,791 patients, 52.55%). Just over half (542,919 patients, 56.41%) were female. Similarly, in patients with HM and IM, most were 40–64 years of age (601 patients, 53.05%) or > 65 years of age (340 patients, 30.01%), and the majority were Caucasian (968 patients, 85.44%) and from the Midwest region (951 patients, 83.94%). As with the overall HM cohort, just over half of patients with HM and IM (644 patients, 56.84%) were female (Table 2).

**Table 2:** Demographic and clinical characteristics of patients with HM and patients with HM and IM

	Overall HM cohort *N* = 962,428		HM and IM cohort *N* = 1133	
	*n*	(%)	*n*	(%)
Age group				
0–12	68,892	7.16	40	3.53
13–17	23,548	2.45	13	1.15
18–39	139,684	14.51	139	12.27
40–64	374,517	38.91	601	53.05
65+	355,453	36.93	340	30.01
Sex				
Female	542,919	56.41	644	56.84
Male	418,637	43.50	489	43.16
Race				
African American	100,958	10.49	84	7.41
Asian	15,613	1.62	14	1.24
Caucasian	757,088	78.66	968	85.44
Other/unknown	88,769	9.22	67	5.91
Ethnicity				
Hispanic	40,235	4.18	52	4.59
Non-Hispanic	826,907	85.92	1035	91.35
Unknown	95,286	9.90	46	4.06
US region				
Midwest	505,791	52.55	951	83.94
Northeast	129,764	13.48	38	3.35
South	203,603	21.16	90	7.94
West	92,147	9.57	40	3.53
Other/unknown	31,123	3.23	14	1.24
Clinical forms of IM				
Pulmonary mucormycosis	–	–	45	3.97
Rhinocerebral mucormycosis	–	–	35	3.09
Gastrointestinal mucormycosis	–	–	0	0.00
Cutaneous mucormycosis	–	–	29	2.56
Disseminated mucormycosis	–	–	12	1.06
Mucormycosis, unspecified	–	–	109	9.62
Other zygomycoses	–	–	8	0.71
Zygomycosis, unspecified	–	–	54	4.77

*HM* hematologic malignancy, *IM* invasive mucormycosis, *N* the total number of patients, *n* number of patients in the category

Of the 1133 patients with HM and IM, clinical forms of mucormycosis (specified and unspecified) were identified by ICD10 code in 292 patients. Of these, the clinical form was specified in 129 patients, with the most common being pulmonary mucormycosis (45 patients, 3.97%). Clinical forms of mucormycosis were not identified in the remaining 841 patients, as these patients were identified only by the ICD9 code 117.7.

### Comorbidities and other diagnoses

The mean CCI score in patients with HM and IM was 3.52 (SD 2.11) post-index. Most patients (1100 patients; 97.09%) had a Charlson Comorbidity Condition. Four patients (0.35%) had HIV/AIDS. The most common comorbidity in the overall HM cohort, based on ICD codes, was hypertension [unspecified essential (243,860 patients, 25.34%) and essential primary (233,997 patients, 24.31%); Table 3]. In addition to hypertension, comorbidities observed in ≥ 15% of patients included other/unspecified hyperlipidemia [166,752 patients, 17.33% (ICD9); 144,349 patients, 15.00% (ICD10)], encounter for immunization [162,653 patients, 16.90% (ICD10)], unspecified anemia [157,597 patients, 16.37% (ICD9)], and other long-term drug therapy [147,499 patients, 15.33% (ICD10); Table 3]. In patients with HM and IM, the most common (*n* > 200) comorbidities and other diagnoses, based on ICD9 and ICD10 codes, included essential primary hypertension (570 patients, 50.31%), unspecified essential hypertension (463 patients, 40.86%), and long-term (current) use of other medications [302 patients, 26.65% (ICD9); 416 patients, 36.72% (ICD10); Table 3]. Diabetes mellitus without complications was observed in 17.92% (ICD9) and 18.80% (ICD10) of patients with HM and IM (Table 3).

**Table 3:** Most common (*n* > 120,000 *for HM*; *n* > 200 *for HM and IM*) post-index comorbidities and other diagnoses, based on ICD9 and ICD10 codes, in patients with HM and in patients with HM and IM

Comorbidity/other diagnosis	ICD code	*n*	(%)
Patients with HM			
Unspecified essential hypertension	ICD9 401.9	243,860	25.34
Essential (primary) hypertension	ICD10 I10	233,997	24.31
Other and unspecified hyperlipidemia	ICD9 272.4	166,752	17.33
Encounter for immunization	ICD10 Z23	162,653	16.90
Anemia, unspecified	ICD9 285.9	157,597	16.37
Other long-term (current) drug therapy	ICD10 Z79.899	147,499	15.33
Hyperlipidemia, unspecified	ICD10 E78.5	144,349	15.00
Long-term (current) use of other medications	ICD9 V58.69	135,159	14.04
Neutropenia, unspecified	ICD9 288.00	134,233	13.95
Encounter for general adult medical examination without abnormal findings	ICD10 Z00.00	123,282	12.81
Anemia, unspecified	ICD10 D64.9	120,963	12.57
Patients with HM and IM			
Essential primary hypertension	ICD10 I10	570	50.31
Unspecified essential hypertension	ICD9 401.9	463	40.86
Malignant neoplasm of connective and other soft tissue	ICD9 171	313	27.63
Long-term (current) use of other medications	ICD9 V58.69	302	26.65
Other long-term (current) drug therapy	ICD10 Z79.899	416	36.72
Encounter for immunization	ICD10 Z23	335	29.57
Other and unspecified hyperlipidemia	ICD9 272.4	300	26.48
	ICD10 E78.5	329	29.04
Encounter for screening mammogram for malignant neoplasm of breast	ICD10 Z12.31	267	23.57
Gastro-esophageal reflux disease without esophagitis	ICD10 K21.9	247	21.80
Mixed hyperlipidemia	ICD9 272.2	246	21.71
	ICD10 E78.2	237	20.92
Esophageal reflux	ICD9 530.81	223	19.68
Type II diabetes mellitus without complications	ICD10 E11.9	213	18.80
Diabetes mellitus without mention of complication type II or unspecified type not stated as uncontrolled	ICD9 250.00	203	17.92

Phycomycosis or mucormycosis (ICD9 117.7) is not listed as a comorbidity

*HM* hematologic malignancy, *ICD* international classification of diseases, *IM* invasive mucormycosis, *n* number of patients in the category

### Treatments

In the overall HM cohort, 119,414 patients were prescribed an antifungal treatment within 360 days post-index. The most commonly prescribed antifungal treatment was fluconazole (109,372 patients, 91.59% of HM patients with antifungal prescriptions).

Of the 119,414 patients with HM who had also been prescribed an antifungal treatment, 382 (0.32% of HM cohort; 33.72% of HM and IM cohort) had IM, while 751 patients (66.28%) with IM received no antifungal treatment (Table 4).

**Table 4:** The antifungal treatment prescribed to patients with hematologic malignancy and invasive mucormycosis

	Overall HM cohort *N* = 962,428		HM and IM cohort *N* = 1133	
	*n*	%	*n*	%
Any antifungal treatment	119,414	12.41	382	33.72
Fluconazole	109,372	11.36	275	24.27
Voriconazole	9657	1.00	137	12.09
Micafungin	9443	0.98	72	6.35
Posaconazole	5080	0.53	185	16.33
Any amphotericin B	3521	0.37	177	15.62
Itraconazole	2685	0.28	24	2.12
Caspofungin	2635	0.27	56	4.94
Anidulafungin	1972	0.20	16	1.41
Isavuconazole	825	0.09	59	5.21

*HM* hematologic malignancy, *IM* invasive mucormycosis, *N* the total number of patients, *n* number of patients in the category

Of the 382 patients with HM and IM who had been prescribed an antifungal agent, the most common were fluconazole (275 patients, 71.99%), posaconazole (185 patients, 48.43%), and AmB (177 patients, 46.34%), indicating that most patients received more than one agent. In the 122 patients coinfected with IM and IA, the most commonly prescribed antifungal agents were AmB (93 patients, 76.23%), posaconazole (86 patients, 70.49%), fluconazole (72 patients, 59.02%), and voriconazole (70 patients, 57.38%), also demonstrating that these patients receive multiple antifungal agents.

## Discussion

In this retrospective, observational study using US patient data from the Optum® EHR dataset, we identified 1133 patients with a diagnosis of HM and IM. Of these patients, most were Caucasian, > 40 years of age, and located in the Midwest region of the USA. It should be noted, however, that the majority of patients in the overall HM cohort in this study were localized in the Midwest, so these characteristics are not necessarily associated with IM. Common comorbidities and other diagnoses included essential hypertension, malignant neoplasm of connective and other soft tissue, long-term use of other medications, encounter for immunization, and hyperlipidemia. The most commonly prescribed antifungal agents in this group were fluconazole, posaconazole, and any AmB.

The 1133 patients that we identified with HM and IM were comparatively large versus those of other studies, in which the number of patients with IM ranged from 41–851 patients [1, 8, 11, 13, 15, 23], suggesting that IM infection could be more common than existing studies show. In contrast to our study, the majority of patients in these studies were European, and the data were pooled either from prior clinical records, mycology surveys, or voluntary case registries. In one global study of 851 individual patient case reports, similar proportions of European (34%), Asian (31%), and North and South American patients (28%) were observed [23], although under-reporting may be occurring in some countries [24]. Notably, the proportion of females with IM (56.84%) was higher in the current study compared with that observed in other studies, in which the proportion of females ranged from 30–40% [11, 13, 15]; however, this could be explained by the fact that there was a higher proportion of female patients than male patients in the overall HM cohort in this study.

Several studies have identified a large number (ranging from 44.00–63.40%) of patients with IM and underlying HM [1, 11, 15]. Because IM is rare and difficult to diagnose, this study focused on patients with HM to enrich the population with true IM infections and reduce the possibility of false IM coding. Other than diabetes mellitus, which was observed in a slightly higher proportion of patients in this study (17.92%) compared with that observed in other studies (4.80–17.10%), the most common comorbidities and other diagnoses in this study were not observed elsewhere [1, 11, 15]. Diabetes mellitus is a known risk factor associated with IM, so it was not surprising that it was observed in a large proportion of patients with IM in our study [25]. The most common non-HM comorbidity in patients with HM and IM was essential primary hypertension (50.31% of patients), which seemed to be a unique finding compared with pooled data studies, although this could be because there was a large proportion of patients with hypertension in the overall HM cohort, and a large proportion of patients of ≥ 65 years of age. Interestingly, however, a history of hypertension has been observed in some individual case studies of patients with IM, suggesting a potential association between hypertension and IM that has not been reported in a larger patient population [26–28]. Additional research into these findings could help to further our understanding of the potential predictors of IM in patients with HM.

Of the patients with HM who were prescribed any antifungal, only 0.32% had IM, which could reflect the challenges associated with the efficient diagnosis and treatment of IM. While AmB is strongly recommended as a first-line therapy for IM [14], only a relatively small percentage of patients with HM and IM in our study (15.62%) received AmB treatment compared with other studies, in which the percentage of AmB-treated patients (monotherapy or in combination with other treatments) ranged from 59.46–72.61%. However, only around one-third of patients with HM and IM received any antifungal treatment and, of these, 46.34% received AmB; this again highlights the challenges associated with the diagnosis and treatment of IM but suggests that AmB is prescribed commonly in the patients who are diagnosed and treated. Aside from AmB, the most commonly prescribed antifungal treatments in our study (patients with HM and IM; patients with HM and IM who received any antifungal, respectively) were the azoles, fluconazole (24.27%; 71.99%), and posaconazole (16.33%; 48.43%), suggesting that many patients received more than one antifungal agent. The high number of fluconazole prescriptions is surprising, given that fluconazole is not mold active [29, 30]. This could be influenced by the fact that fluconazole and posaconazole are often used as long-term prophylactic treatments in patients with neutropenia [9, 14]. Prophylactic treatment strategies may lead to overuse of antifungals, causing unnecessary exposure and the potential development of resistance, as well as an increased cost burden [31]. Antifungal stewardship (AFS) programs can be utilized to monitor and intervene in antifungal treatment strategies, although cultural and professional influences may present a barrier to the usefulness of AFS [31, 32]. The data presented here may help to illustrate large-scale prescribing behaviors, although more research is necessary to further our understanding of antifungal prescribing behaviors and reduce inappropriate antifungal use.

The frequency of azole use may have been associated with the prevention or treatment of IA. Posaconazole, for example, is recommended for salvage treatment and prophylaxis against IA [30]. However, some evidence suggests that prophylactic azoles do not prevent IM [33] and have been associated with breakthrough IM infections in some patients [11, 15]. Although coinfection with IA and IM is considered rare [34], it was observed in 14.21% of the patients with HM and IM in the current study, suggesting that it may be more common than previous patient data have indicated. Further study and increased clinical awareness of IA and IM coinfection may lead to improved diagnosis and treatment in the coming years.

This study was descriptive only, with observational data, and yielded a high-level epidemiologic view. No statistical inference testing was performed, and no outcomes data, including survival, were available; therefore, further study is required to draw meaningful conclusions from the data. There are inherent limitations with large patient datasets and how the data are collected and recorded. For example, the HM cohort mainly includes patients localized in the Midwest, which may not represent the wider population of IM patients. There were also more data available in recent years compared with earlier years, suggesting an increase over time in the number of patients with data recorded in EHR datasets. Additionally, the data were not collected for research purposes, so it may be more difficult to analyze compared with clinical data. For example, Mucorales species and the method of diagnosis could not be extracted from the data. Finally, diseases could have been miscoded, and it was not possible to retrospectively confirm diagnoses from the deidentified patient data. Despite the inherent limitations of analyzing large patient datasets, the potential benefit of this approach has been shown in this study. However, these limitations should be taken into account in future studies.

## Conclusion

In conclusion, this study represents a promising new approach for analyzing large cohorts of patients with apparently rare diseases, which are challenging to identify prospectively. With further study, the potential applications of large dataset analysis for patients with IM include identifying risk factors, improving diagnosis, and assessing current treatment practices and outcomes. Implementation of research standards, harmonized guidance and more sophisticated methods will facilitate the continuation of informative research in future real-world studies.

## Data Availability

The data that support the findings of this study are available from Optum® but restrictions apply to the availability of these data, which were used under license for the current study, and so are not publicly available. The license allows Pfizer to access currently available data from the Optum® deidentified EHR dataset, which is subject to change over time in line with changes to the network. Data are however available from the authors upon reasonable request and with permission of Optum®. Please contact the corresponding author of this manuscript (Yayue Zhang, zhangyayue@gmx.com) or Optum® directly (Yunwei.sun@optum.com) if you would like to request data, or require any further information.

## Abbreviations

AmB: Amphotericin B
AFS: Antifungal stewardship
AML: Acute myeloid leukemia
CCI: Charlson Comorbidity Index
EHR: Electronic health record
HM: Hematologic malignancy
IA: Invasive aspergillosis
ICD: International classification of diseases
IM: Invasive mucormycosis
*N*: The total number of patients
*n*: Number of patients in the category
